# The effect of non-surgical and surgical mechanical root debridement on infrabony defects: a retrospective study

**DOI:** 10.1038/s41598-021-99205-z

**Published:** 2021-10-06

**Authors:** Jad Majzoub, Ali Salami, Shayan Barootchi, Lorenzo Tavelli, Hsun-Liang Chan, Hom-Lay Wang

**Affiliations:** 1 Department of Periodontics and Oral Medicine, University of Michigan School of Dentistry, 1011 North University Avenue, Ann Arbor, MI 48109-1078 USA; 2 Department of Mathematics, Faculty of Sciences, Lebanese University, Nabatieh, Lebanon; 3 Division of Periodontology, Department of Oral Medicine, Infection and Immunity, Harvard School of Dental Medicine, Boston, MA USA

**Keywords:** Diseases, Health care

## Abstract

The aim of this retrospective study was to assess the outcomes of non-surgical and surgical mechanical root debridement for the treatment of infrabony defects and explore potential prognostic factors. Treated infrabony defects followed for at least 1 year were selected. All data pertaining to the clinical outcomes were recorded. Multi-level regression analysis and Cox Proportional-Hazards Models were used to assess the immediate (3–6 months) clinical outcomes, survival of the treated teeth, and factors influencing these results. 132 patients were included in the analysis. The analysis showed 1.42 ± 1.71 and 2.23 ± 1.64 mm in pocket depth (PD) reduction, 0.13 ± 1.83 and 0.08 ± 1.76 mm in clinical attachment level (CAL) gain, and 1.29 ± 1.56 and 2.15 ± 1.33 mm increase in gingival recession (REC) for the non-surgical and surgical groups, respectively. The 5-year survival rates were 93% for the non-surgically and 90% for the surgically treated teeth. Several factors affected clinical outcomes and tooth survival. Within its limitations, the treatment of infrabony defects with non-surgical and surgical mechanical root debridement was found to result in moderate but significant PD reduction, nevertheless, this may also be attributable to the resultant REC.

## Introduction

Periodontitis is a complex multifactorial disease that often leads to the formation of infrabony defects^1^. If untreated, these defects can increase the risk for the disease progression by 10-fold^2^, leading to eventual tooth loss^1^. Furthermore, the treatment of infrabony defects poses many clinical challenges, making its presence one of the influential factors when determining the complexity of periodontal disease^3^.

A large number of studies have evaluated the effect of periodontal regeneration for infrabony defects and shown positive clinical and radiographic outcomes^4–12^, as well as histological evidence of new cementum, periodontal ligament and alveolar bone regeneration^13,14^. Nevertheless, this treatment modality presents some challenges, such as additional material costs^4^, patient morbidity^15^, and being technique sensitive. In addition, factors such as smoking, the infrabony defect morphology, surgical technique considerations (e.g., incision design) as well as post-surgical exposure of the regenerative materials can substantially impact the results of the regenerative treatment outcomes^7,16,17^.

Despite the presence of many clinical studies evaluating treatment of infrabony defects with guided tissue regeneration (GTR) or biologics agents, fewer studies have looked into the treatment outcomes of surgical (open flap debridement, OFD) and non-surgical (scaling and root planing, SRP) mechanical root debridement alone^18^. Moreover, the majority of studies that have evaluated the effect of SRP on infrabony defects were conducted in a population with a limited sample size^18–21^, had a relatively short follow-up^18–22^ and reported considerable ﻿heterogeneity in their treatment outcomes^23^.

With the increase in popularity of ﻿simplified periodontal procedures that provide less chair-side time and surgical equipment, as well as reduced treatment costs and patient morbidity^23^, investigating the benefit of surgical and non-surgical mechanical debridement alone for periodontal defects is crucial. Therefore, the aims of this study were to evaluate the clinical outcomes and survival of teeth associated with infrabony defects treated with non-surgical or surgical mechanical debridement alone, and to assess potential factors affecting the results. Specifically, the following study aims were examined: (1) the immediate short-term (3–6 months) clinical outcomes following active periodontal therapy (APT), (2) the survival of the treated teeth (> 1 year following APT), and (3) the factors influencing both the immediate treatment outcomes, and the survival of the treated teeth with periodontal infrabony defects.

## Materials and methods

### Study design

The current investigation was designed according to the principles presented in the Helsinki Declaration of 1975, as revised in 2013 for biomedical research involving human patients. The study was approved by the Institutional Review Board for Human Studies, School of Dentistry, University of Michigan, Ann Arbor, MI (HUM00186895) to be conducted at the Department of Periodontology and Oral Medicine within the same institution. The same IRB has waived the need for obtaining informed consent from patient based on the EXEMPTION 4(iii) at 45 CFR 46.104(d): secondary research for which consent is not required.

This retrospective study selected all patients that had undergone APT including non-surgical mechanical debridement alone or followed by surgical mechanical root debridement for infrabony defects followed by supportive periodontal therapy (SPT), between January 1980 and December 2018 at the School of Dentistry, University of Michigan, Ann Arbor, MI.

All paper files and digital charts of patients with treated infrabony defects with non-surgical or surgical mechanical debridement were carefully scanned and analyzed by two independent and pre-calibrated investigators (JM, SB). At every stage, after examining the gathered data, in case of a disagreement, discussion was held by the two reviewers to reach consensus. If resolution was not possible, a senior author (either H-LC or HLW) was consulted and their decision was decisive. ﻿The current research was prepared in compliance with the STROBE guidelines (www.strobe-statement.org, see Supplementary Table S1).

### Eligibility criteria

The present study included patients having a tooth associated with an infrabony defect (at least 3 mm of radiographic defect depth^24,25^), that underwent a phase of APT consisting of either (1) non-surgical mechanical debridement alone (i.e., SRP), or (2) SRP followed by re-evaluation and then surgical mechanical debridement (i.e., OFD). All OFD cases were achieved by access flap without papilla preservation. In all cases, both hand and ultrasonic instruments were used. After completion of the APT phase, all patient entered a SPT phase (a minimum of 1 visit/year). All subject records must have had at least 1 year of follow-up following the completion of APT and with complete records of clinical data. In addition, subjects must have received annual SPT at least 1 visit per year. In cases where a single subject had more than one tooth associated with a treated infrabony defect, a single tooth was randomly selected.

For all the included population, treatment was performed by periodontal post-doctoral residents. APT consisted of verbal individualized oral hygiene instructions^26^, followed by supra- and sub-gingival SRP. Following SRP, all patients presented for a re-evaluation appointment (4–6 weeks) during which it was determined whether further surgical therapy, i.e., OFD, was needed. OFD was then undergone when needed. The baseline was defined as the last day of the active treatment phase, i.e., last round of SRP in APT for patients receiving non-surgical therapy only, or the day of the surgery for patients that underwent OFD surgery. Patients that received regenerative therapy or root resection, as their APT treatment or during SPT or that had more than one OFD surgery at the infrabony defect area were excluded from the study.

### Data collection and classification

The following information were obtained for all qualified patients: (1) patient demographic information (age, gender, etc.); (2) medical history (i.e. smoking, diabetes, etc.); (3) location of the treated tooth; (4) clinical parameters of the infrabony defect: pocket depth (PD), gingival recession (REC) and clinical attachment level (CAL) at baseline and at the 3- to 6-month re-evaluation; (5) type of intervention for the APT (SRP/OFD); (6) association of the treated tooth with a furcation defect (yes/no); (7) tooth type (single-rooted/multi-rooted); (8) follow-up time (until tooth extraction or the last maintenance appointment); and (9) frequency of maintenance visits throughout the SPT.

### Study outcomes

#### Survival

Survival, as the primary outcome of this study was assessed via the Kaplan–Meier analysis. The last documented follow-up visit of a patient and the included tooth was considered as evidence for its presence and survival up to that time point. Teeth that were extracted due to periodontal reasons (e.g., poor periodontal status, tooth mobility, suppuration, periodontal abscess) were considered as “failures” and an “event” in the Kaplan–Meier analysis. In a clinical scenario where a tooth was extracted due to other reasons (e.g., restorative, endodontic or prosthodontic reasons), this tooth was excluded from the study, therefore not contributing to the failure/survival rate of the cohort, as the outcome was failure by periodontal reasons specifically. Furthermore, as with previous reports, the potential influence of several variables on tooth survival was assessed^27^.

#### Immediate clinical outcomes of APT

The changes in the clinical parameters (PD, CAL, REC) were compared from baseline to the 3–6 months evaluation. Additionally, the influence of other recorded variables was assessed on the treatment outcomes.

### Data management and statistical analysis

Descriptive statistics were carried out and reported as frequencies and percentages for categorical variables and as means ( ±) standard deviation (SD) for continuous ones. Normality was assessed using the Kolmogorov–Simonov test. The changes in CAL, PD, and REC from baseline to the 3- to 6-month evaluation were assessed using dependent *t *tests when data was normally distributed. Otherwise, Wilcoxon signed-rank test was used.

Baseline comparisons between groups (OFD vs. SRP) were performed using independent t test when data was normally distributed. Otherwise, Wilcoxon rank-sum test was conducted. The chi-square test was used to assess any significant difference between the categorical variables.

Mixed-effects uni- and multi-level regression analyses were performed to identify predictive factors for CAL, PD, and REC at the 3- to 6-months evaluation timepoint. Kaplan–Meier survival probabilities were calculated, and the curves for the entire follow-up period were subsequently plotted.

Multi-variate Cox Proportional Hazard models and step-wise regression analyses performed (using likelihood ratio tests) were used for assessing correlations between independent variables and tooth loss. Hazard ratios (HR) and corresponding 95% confidence intervals (CI) were generated, and a p value threshold of 0.05 was set for all significant testing. All analyses were performed using SPSS software (IBM Corp. Released 2019, SPSS Statistics for Windows Version 26.0, Armonk, NY), while the plots were generating using two other software [Origin software (OriginPro, Version 2019b. OriginLab Corporation, Northampton, MA, USA) and Rstudio (Version 1.1.383, RStudio, Inc., Boston, Massachusetts, USA)], using the survminer^28^, survival^29^, and ggplot2^30^ packages.

## Results

### Study population

A total of 132 patients [64 males and 68 females, 92 receiving SRP alone (mean age: 51.90 ± 15.26 years) and 40 receiving SRP followed by OFD (mean age: 53.35 ± 11.88 years)] were included in this study (Supplementary Figure S1). The mean follow-up for the selected cases in the SRP group was 5.21 ± 3.12 years (range 1–14.5 years) and in the OFD group was 6.02 ± 3.51 years (range 1–20.25 years). The average SPT visits for the included patients in the SRP group was 1.95 ± 0.58 times/year and in the OFD group was 1.97 ± 0.73 times/year. ﻿The characteristics of the subject sample at baseline are presented in detail in Supplementary Table S2.

### Survival analysis

**Figure 1:**
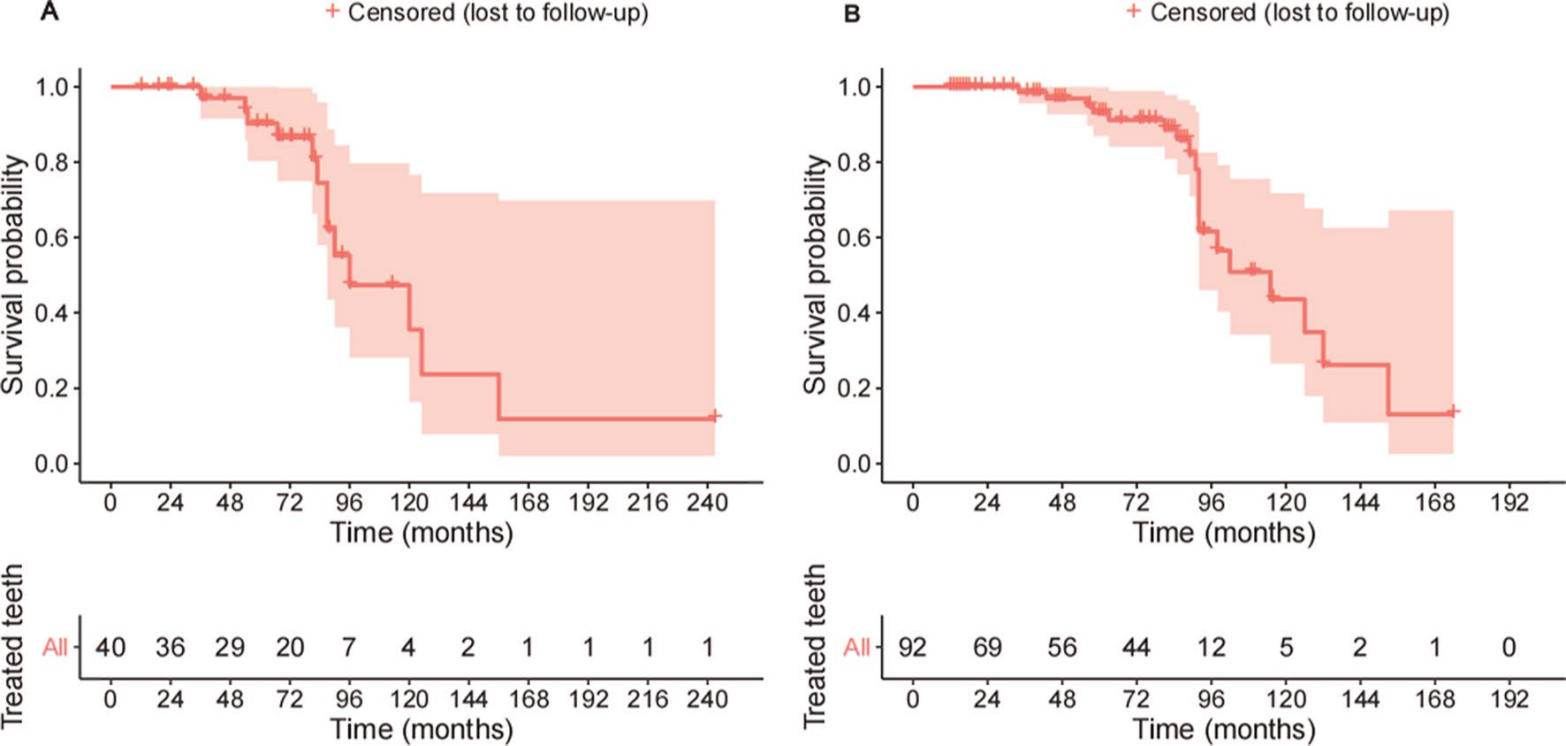
Kaplan–Meier survival curve for the entire follow-up period for the OFD (**A**) and the SRP (**B**) groups. Each event represents a tooth loss. The reddish hue represents the upper and lower limit of the 95% confidence bands.

From baseline until the final gathered follow-up appointment, a total of 19 teeth were lost in the SRP group and 13 teeth in the OFD group. For the SRP group, the 5- and 10-year survival rates were 93% and 43%, respectively. For the OFD group, the 5- and 10-year survival rates were 90% and 35%, respectively. Figure 1 demonstrates the survival curves of the treated teeth. The life table analysis presents the number of followed, censored, and extracted teeth at the respective time points (Supplementary Table S3).

#### SRP group

Results from the univariate analysis of the cox proportional hazard models, evaluating the influence of potential variables on tooth survival are presented in Table 1. Teeth that were affected by both an infrabony and a furcation defect had significantly lower survival rates. In addition, teeth that had deeper PD at the 3- to 6-month evaluation appointment also affected survival rates negatively. However, patients who attended more SPT visits per year had lower risk for tooth loss.

**Table 1:** Results of the multilevel cox proportional hazard models evaluating the effect of different variables on the survival of the treated teeth.

Variable	Univariate analysis				Multivariate analysis			
	HR	Std. error	95% CI	p value	HR	Std. error	95% CI	p value
**OFD**								
Age	0.999	0.026	(0.949, 1.052)	0.963				
Gender (male)	1.492	0.622	(0.441, 5.051)	0.520				
Smoking	2.369	0.650	(0.663, 8.469)	0.184				
Diabetes	0.577	1.058	(0.073, 4.592)	0.603				
**Maintenance per year**	**0.187**	**0.726**	**(0.045, 0.776)**	**0.021**	0.233	0.265	(− 0.306, 0.771)	0.386
Baseline PD	1.008	0.170	(0.723, 1.407)	0.962				
Baseline CAL	1.279	0.184	(0.891, 1.834)	0.182				
**Re-evaluation PD**	**1.662**	**0.184**	**(1.160, 2.381)**	**0.006**	**− 0.584**	**0.251**	**(**− **1.093, − 0.075)**	**0.026**
**Re-evaluation CAL**	**1.586**	**0.144**	**(1.195, 2.105)**	**0.001**	**0.625**	**0.177**	**(0.266, 0.985)**	**0.001**
**Association with furcation defects (yes)**	**3.342**	**0.602**	**(1.027, 10.872)**	**0.045**	− 0.046	0.723	(− 1.514, 1.423)	0.950
Type of tooth (multi-rooted)	0.444	0.646	(0.125, 1.576)	0.209				
**SRP**								
Age	1.014	0.019	(0.978, 1.053)	0.447				
Gender (male)	0.887	0.468	(0.354, 2.219)	0.797				
Smoking	1.607	0.478	(0.630, 4.102)	0.321				
Diabetes	1.824	0.761	(0.410, 8.111)	0.430				
**Maintenance per year**	**0.224**	**0.523**	**(0.081, 0.625)**	**0.004**	**0.316**	**0.528**	**(0.112, 0.890)**	**0.029**
Baseline PD	0.810	0.200	(0.547, 1.200)	0.293				
Baseline CAL	1.008	0.121	(0.796, 1.277)	0.947				
**Re-evaluation PD**	**1.444**	**0.160**	**(1.056, 1.975)**	**0.021**	0.748	0.252	(0.456, 1.225)	0.248
Re-evaluation CAL	1.163	0.127	(0.907, 1.492)	0.234				
**Association with furcation defects (yes)**	**12.934**	**0.540**	**(4.489, 37.263)**	** < 0.001**	**23.401**	**0.980**	**(3.431, 159.611)**	**0.001**
Type of tooth (multi-rooted)	0.680	0.464	(0.274, 1.686)	0.405				

The values in bold signifies statistical significance; CI, confidence intervals. Data related to the presented variables was available for all 132 subjects.

Multi-variate analyses revealed that the average number of SPT visit/year (0.316 (95% CI [0.112, 0.89], p = 0.029)), and the association with furcation defects (23.401 (95% CI [3.431, 159.611], p = 0.001)) both significantly affected the survival in the multi-variate model. When the association with furcation defect variable was excluded from the model, multi-variate analyses revealed that the average number of SPT visit/year (0.235 (95% CI [0.086, 0.644], p = 0.005)), and the PD obtained at the 3–6 months evaluation (1.407 (95% CI [1.037, 1.910], p = 0.028)) both significantly affected the survival in the multi-variate model (Supplementary Table S4).

**Figure 2:**
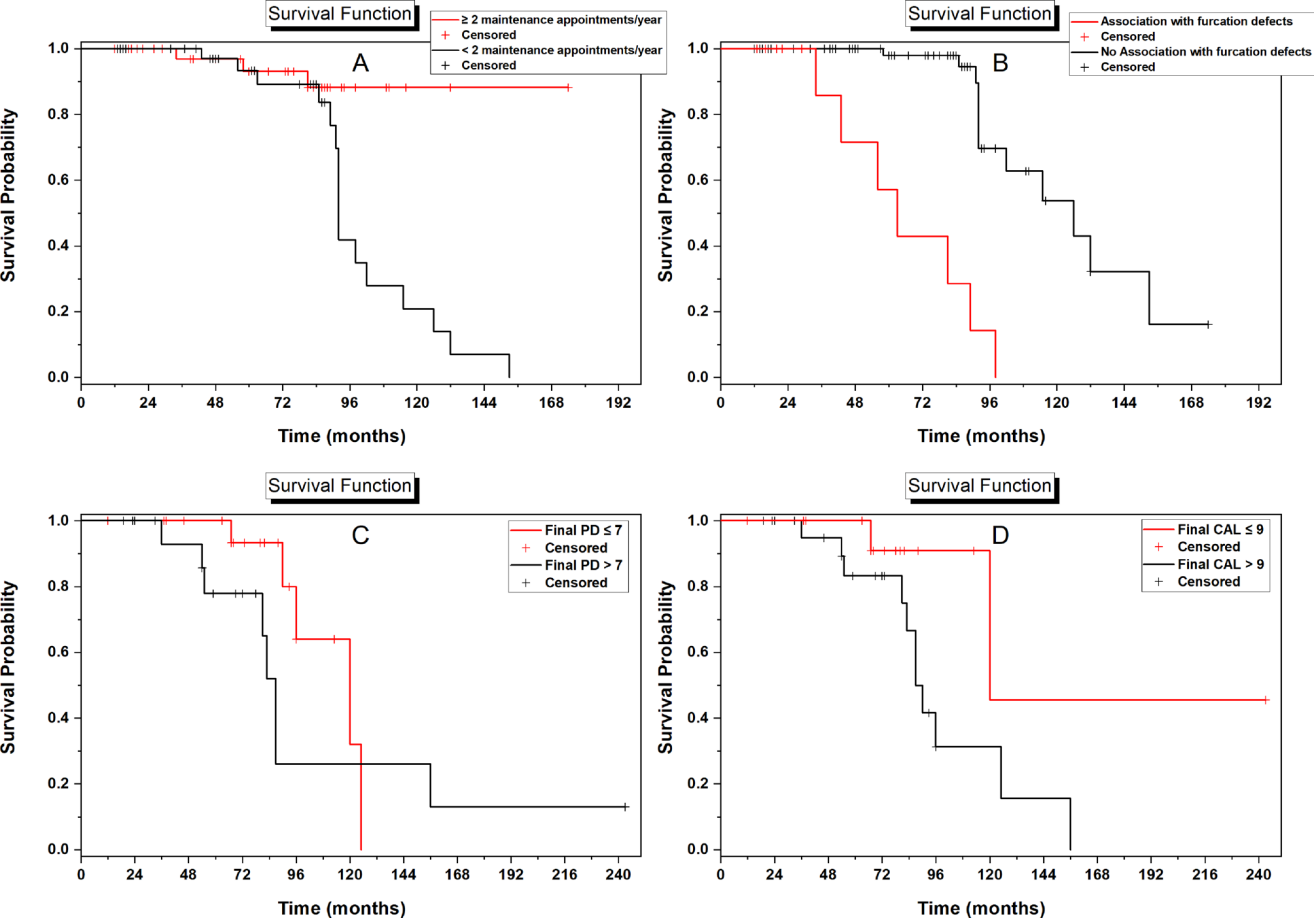
Kaplan–Meier survival curves displaying the comparison between: (**A**) teeth treated with SRP, receiving at least 2 maintenance appointments/year (red) and less than 2 maintenance appointments/year (black); (**B**) teeth treated with SRP, associated (red) versus not associated with furcation defects (black); (**C**) teeth treated with OFD, having at most 7 mm PD (red) and more than 7 mm PD (black) at the 3–6 months evaluation; and (**D**) teeth treated with OFD, having at most 9 mm CAL (red) and more than 9 mm CAL (black) at the 3–6 months evaluation. The median cut off value of PD and CAL were used when dichotomizing the stratifying variable. Event = Tooth loss.

Figure 2A illustrates the different survival curves for teeth receiving < 2 versus ≥ 2 SPT visits per year and for teeth associated versus not associated with furcation defects. Figure 2B presents survival curves for treated associated and not associated with furcation defects. The Hazard ratios (HR) and 95% confidence intervals (CI) of tooth loss are presented in Table 1.

#### OFD group

Univariate analysis showed that teeth that were affected by both an infrabony and a furcation defect had significantly lower survival rates. In addition, teeth that had deeper PD and higher CAL at the 3- to 6-month evaluation appointment also affected survival rates negatively. However, patients who attended more SPT visits per year had lower risk for tooth loss.

Multi-variate analyses revealed that the PD obtained at the 3–6 months evaluation (− 0.584 (95% CI [− 1.093, − 0.075], p = 0.026)) as well as the CAL obtained at the 3–6 months evaluation (0.625 (95% CI [0.266, 0.985], p = 0.001)) both significantly affected the survival in the multi-variate model.

Figure 2C,D illustrates the different survival curves for teeth according to the PD and CAL, respectively, obtained at the 3–6 months evaluation. The Hazard ratios (HR) and 95% confidence intervals (CI) of tooth loss are presented in Table 1.

### Clinical outcomes

#### SRP group

At baseline, 48.9% of sites presented with BOP, a mean PD of ﻿8.11 ± 1.63 mm, REC of 0.67 ± 1.51 mm, and CAL of 8.78 ± 2.07 mm. At the 3- to 6-month evaluation, BOP had been reduced to 9.8%. In addition, PD reduction of 1.42 ± 1.71 mm as well as an increase in REC of 1.29 ± 1.56 mm were observed, and both changes were statistically significant (p < 0.001). In addition, a CAL gain of 0.13 ± 1.83 mm was also observed (p = 0.569).

#### OFD group

Prior to the APT phase, 60% of sites presented with BOP, a mean PD of ﻿9.65 ± 1.54 mm, REC of 0.43 ± 1.22 mm, and CAL of 10.08 ± 1.73 mm. At the 3- to 6-month evaluation, BOP had been reduced to 17.5%. A statistically significant PD reduction of 2.23 ± 1.64 mm as well as an increase in REC of 2.15 ± 1.33 mm were also observed (p < 0.001). In addition, a CAL gain of 0.08 ± 1.76 mm was witnessed (p = 0.709).

When the defects were divided based on whether or not a mucoperiosteal flap (SRP *versus* OFD groups) was elevated, a non-statistically significant difference in CAL gain was observed between the groups (p = 0.86). However, a statistically significant increase in REC (p = 0.001) and PD reduction (p = 0.01) were observed in the OFD group.

### Factors affecting the clinical outcomes

#### Clinical attachment level gain:

Table 2A–C depict the results of the regression models in detail. When evaluating the potential factors affecting CAL gain in the SRP group, univariate analyses (Table 2A) showed that initial CAL, and a combination of infrabony and furcation defect on the same tooth significantly and negatively affected the outcomes.

**Table 2:** Results of the regression models evaluating the effect of different variables on the CALs (2A), REC (2B) and PD (2C) of the treated defects at the 3–6 months re-evaluation appointment.

Variable	Univariate analysis				Multivariate analysis			
	Estimate	Std. error	95% CI	p value	Estimate	Std. error	95% CI	p value
(A) **OFD**								
Age	0.048	0.025	(− 0.003, 0.098)	0.065				
Gender (male)	0.544	0.558	(− 0.585, 1.673)	0.335				
Smoking	− 0.225	0.590	(− 1.419, 0.969)	0.705				
Diabetes	1.480	0.752	(− 0.042, 3.003)	0.056				
Baseline PD	0.108	0.184	(− 0.264, 0.480)	0.561				
Baseline CAL	0.246	0.160	(− 0.078, 0.570)	0.132				
Association with furcation defects (yes)	− 1.457	0.819	(− 3.115, 0.201)	0.083				
**SRP**								
Age	0.013	0.013	(− 0.011, 0.038)	0.285				
Gender (male)	0.497	0.381	(− 0.259, 1.254)	0.195				
Smoking	0.531	0.413	(− 0.289, 1.352)	0.202				
Diabetes	0.074	0.846	(− 1.607, 1.754)	0.931				
Baseline PD	0.110	0.117	(− 0.123, 0.343)	0.352				
**Baseline CAL**	**0.469**	**0.079**	**(0.312, 0.625)**	** < 0.001**	**0.478**	**0.075**	**(0.329, 0.628)**	** < 0.001**
**Association with furcation defects (yes)**	**− 1.300**	**0.553**	**(− 2.398, − 0.202)**	**0.021**	**− 1.420**	**0.461**	**(− 2.336, − 0.503)**	**0.003**

Variable	Univariate analysis				Multivariate analysis			
	Estimate	Std. error	95% CI	p value	Estimate	Std. error	95% CI	p value
(B) **OFD**								
Age	− 0.034	0.017	(− 0.069, 0.001)	0.060				
Gender (male)	− 0.516	0.419	(− 1.364, 0.331)	0.225				
Smoking	− 0.451	0.441	(− 1.343, 0.442)	0.313				
Diabetes	− 0.176	0.596	(− 1.384, 1.031)	0.769				
**Baseline PD**	**0.409**	**0.123**	**(0.160, 0.658)**	**0.002**	**0.409**	**0.123**	**(0.160, 0.658)**	**0.002**
Baseline CAL	− 0.012	0.125	(− 0.265, 0.240)	0.921				
Association with furcation defects (yes)	0.286	0.643	(− 1.016, 1.587)	0.659				
**SRP**								
Age	− 0.006	0.011	(− 0.027, 0.016)	0.593				
Gender (male)	− 0.071	0.327	(− 0.721, 0.580)	0.829				
Smoking	− 0.268	0.354	(− 0.971, 0.435)	0.451				
Diabetes	− 0.099	0.721	(− 1.531, 1.333)	0.891				
**Baseline PD**	**0.556**	**0.082**	**(0.394, 0.718)**	** < 0.001**	**0.556**	**0.082**	**(0.394, 0.718)**	** < 0.001**
Baseline CAL	− 0.072	0.079	(− 0.228, 0.085)	0.365				
Association with furcation defects (yes)	− 0.625	0.481	(− 1.580, 0.330)	0.197				

Variable	Univariate analysis				Multivariate analysis			
	Estimate	Std. error	95% CI	p value	Estimate	Std. error	95% CI	p value
(C) **OFD**								
Age	0.016	0.022	(− 0.029, 0.061)	0.483				
Gender (male)	0.028	0.526	(− 1.038, 1.093)	0.958				
Smoking	− 0.676	0.540	(− 1.769, 0.417)	0.218				
Diabetes	1.304	0.705	(− 0.123, 2.731)	0.072				
Baseline PD	**0.517**	**0.150**	**(0.213, 0.822)**	**0.001**	**0.517**	**0.150**	**(0.213, 0.822)**	**0.001**
Baseline CAL	0.234	0.149	(− 0.068, 0.536)	0.125				
Association with furcation defects (yes)	− 1.171	0.772	(− 2.733, 0.391)	0.137				
SRP								
Age	0.008	0.012	(− 0.016, 0.031)	0.514				
Gender (male)	0.427	0.357	(− 0.282, 1.135)	0.235				
Smoking	0.263	0.389	(− 0.509, 1.036)	0.500				
Diabetes	− 0.025	0.791	(− 1.597, 1.547)	0.975				
Baseline PD	**0.666**	**0.085**	**(0.497, 0.835)**	** < 0.001**	**0.568**	**0.105**	**(0.360, 0.775)**	** < 0.001**
Baseline CAL	**0.397**	**0.076**	**(0.246, 0.548)**	** < 0.001**	0.101	0.082	(− 0.063, 0.264)	0.225
Association with furcation defects (yes)	**− 1.925**	**0.493**	**(− 2.903, − 0.947)**	** < 0.001**	**− 1.879**	**0.365**	**(− 2.604, − 1.154)**	** < 0.001**

The values in bold signifies statistical significance; CI, confidence intervals. Data related to the presented variables was available for all 132 subjects.

The multivariate analysis revealed that infrabony defects associated with higher initial CAL were associated with increased CAL gain (0.478 (95% CI[0.329, 0.628], p < 0.001) while teeth that were affected by both an infrabony and furcation defect were significantly associated with lower CAL gain (− 1.420 (95% CI [− 2.336, − 0.503], p = 0.003).

None of the factors evaluated in the OFD group seemed to significantly affect the CAL gain witnessed at the 3–6 months re-evaluation appointment.

#### Increase in gingival recession

Table 2B presents the results from the uni- and multi-variate analysis evaluating the effect of different variables on the REC of the treated defects at the 3–6 months re-evaluation appointment.

Results from our univariate analyses showed that cases that presented with higher initial PD were significantly affected by more REC in both SRP and OFD groups.

**Figure 3:**
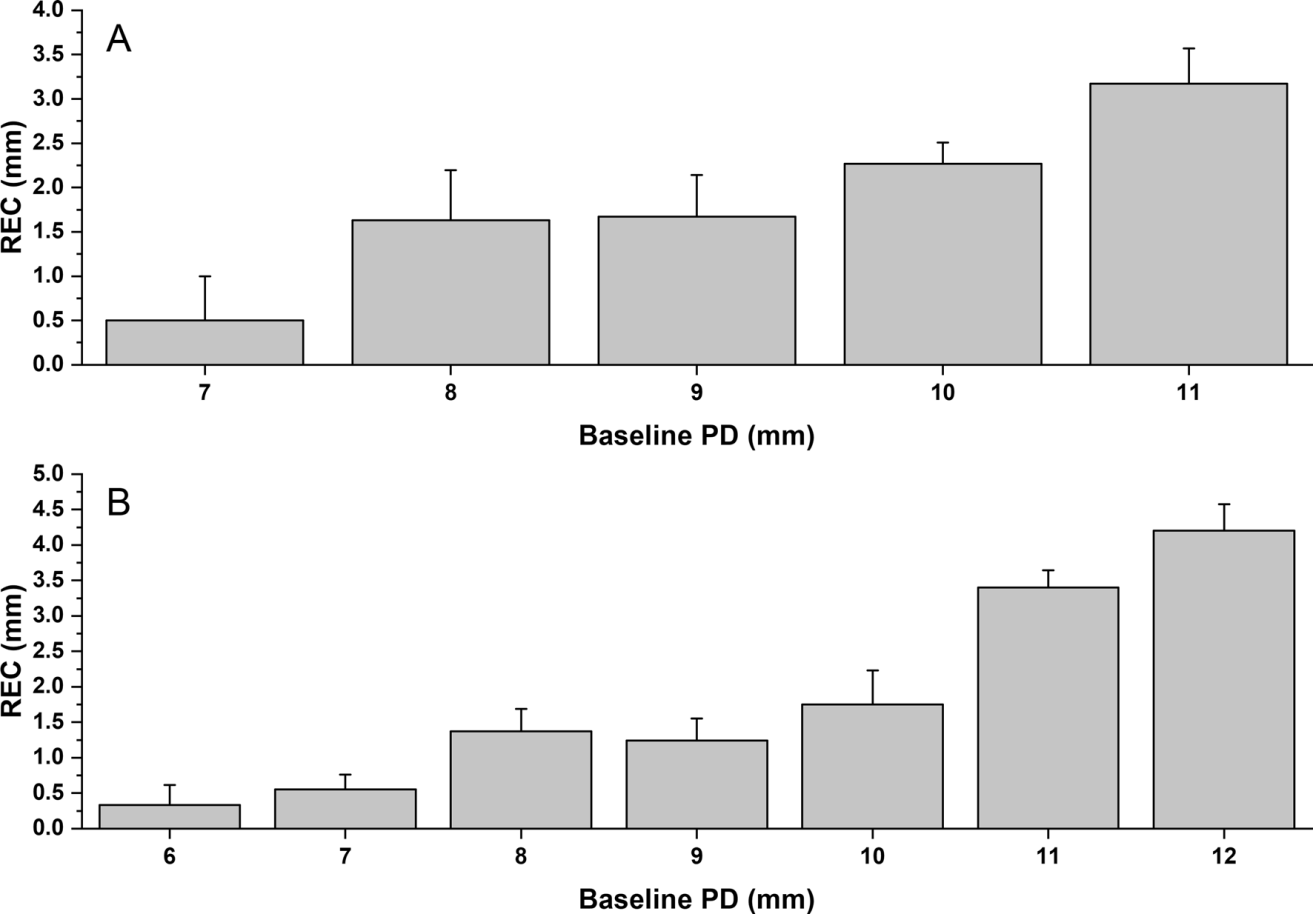
Relationship between the baseline PD and the amount of REC observed at the 3–6 months re-evaluation appointment in the OFD (**A**) and SRP (**B**) groups.

In the multivariate model, we observed that the initial PD factor remained significant for both groups, such that, infrabony defects that had deeper PD experienced more REC in the SRP (0.556 (95% CI [0.394, 0.718], p < 0.001) and OFD (0.409 (95% CI [0.160, 0.658], p = 0.002) groups. Figure 3 illustrates the relationship between baseline PD and the amount of experienced REC at the 3- to 6-month evaluation.

#### Pocket depth reduction

The results of PD reduction from the uni- and multi-variate analysis are presented in Table 2C. Univariate analysis evaluating the predictors of PD reduction found that initial levels of CAL and PD, as well as the association with furcation defects all influenced the outcomes in the SRP group. Initial PD (0.568 (95% CI [0.36, 0.775], p < 0.001) and the association with furcation defects (− 1.879 (95% CI [− 2.604, − 1.154], p < 0.001) both remained significant in the multivariate analysis.

In the OFD group, univariate analysis found that initial levels of PD influenced the outcomes. This factor remained significant in the multivariate analysis (0.517 (95% CI [0.213, 0.822], p < 0.001).

## Discussion

The present study demonstrated that ﻿non-surgical and surgical mechanical debridement of teeth associated with infrabony defects result in moderate significant PD reduction [mean average: 1.42 mm (SRP group); 2.23 mm (OFD group)]. This might have a positive impact on tooth survival, since teeth with deep PD have been shown to have lower survival rates^31,32^. However, this reduction is probably primarily resulting from the amount of recession observed [mean average: 1.29 mm (SRP group); 2.15 mm (OFD group)] since only a small amount of CAL gain [mean average: 0.13 mm (SRP group); 0.08 mm (OFD group)] was noted. These findings corroborate with other studies ﻿that evaluated the effect of SRP and OFD for the treatment of infrabony defects^20,21^. Isidor and colleagues, reported an average of ﻿3.2 mm REC of teeth with infrabony defects treated with OFD and 1.8 mm when treated with SRP alone^20^. In the Isidor study, the PD reduction that occurred was mainly﻿ due to recession of the gingival margin and only to a less extent, as a result of CAL gain. Nonetheless, they observed higher amount of CAL gain compared to the present study (0.9 ± 0.5 mm and 1.6 ± 0.5 mm for OFD and SRP, respectively). This might be due to the more strict recall protocol established in Isidor et al. study where subjects were seen ﻿every two weeks for professional tooth cleaning^20^. When conducting a systematic review and meta-analysis of randomized clinical trials evaluating the clinical performance of conservative surgery on infrabony defects, Graziani et al. showed a CAL gain of 1.65 mm, a PD reduction of 2.85 mm and a REC of 1.15 mm one year following the surgery. Although the Graziani study found REC levels that are comparable to the ones found in the present study, higher levels of CAL gain were achieved in their study. This might be partially attributed to the ﻿types of surgical flap included in their study such as the papilla preservation design that achieved better outcomes when compared to access flaps, as well as the clinician’s experience level^33^.

When investigating potential factors affecting the clinical outcomes, it seems that higher baseline CAL and PD were associated with more CAL gain (SRP group) and PD reduction (both groups), respectively. This is attributed to the reduction of gingival swelling and inflammation and tissue shrinkage after treatment^34^. Conversely, infrabony defects teeth compromised with an additional furcation defect resulted in considerably less PD reduction and CAL gain in the SRP group. Similarly, Nibali and colleagues in a retrospective study reported an average of 2.3 mm PD reduction and 1.5 mm CAL gain 12 to 18 months following treatment in 126 infrabony defects which 29% of them were associated with furcation defects^22^. However, this specific study did not elaborate if the presence of furcation defects influenced the treatment outcomes. Nonetheless, a recent study evaluated the effect of periodontal regeneration using ﻿enamel matrix derivative and papilla preservation flaps on teeth with combined infrabony and furcation defects, showed a substantial clinical improvement^35^. By assessing evidence from literature as well as the findings form this study, we believe that periodontal regeneration may be more appropriate to manage teeth with combined infrabony and furcation periodontal defects. In addition, that the loss of this type of teeth in the present study could be attributed to the presence of furcation defects and not infrabony defects alone. Details on the morphology of the furcation defects included in the present study is presented in Supplementary Table S2.

It is worth noting that results from the present study showed that the 10-year survival rate of teeth associated with infrabony defects and treated with non-surgical and surgical mechanical root debridement was 43% in the SRP group and 35% in the OFD group. Although this rapid drop of the survival rate can be allocated to the smaller remaining sample size at 10 years (Please refer to the life table analysis presented in Supplementary Table S3), angular bony defects have been shown to highly ﻿increase the risk of progressive periodontitis, eventually leading to tooth loss^2^. Another interesting finding from our regression models was that the CAL and PD obtained following APT significantly affected tooth survival in the OFD group. Conversely, baseline CAL and PD was not found to influence tooth survival. This signifies that a successful APT resulting in CAL gain and PD reduction may in fact prolong the lifespan of an infrabony-associated defect. A more frequent SPT program seemed to also prolong tooth survival in the SRP group (Fig. 2A).

In the present study, a relatively low level of BOP was witnessed at baseline, this might be attributed to the effect of smoking on periodontal vasculature^36^, since 31.8% of the subjects included in the present study were smokers. Nonetheless, a remarkable decrease in BOP was witnessed following the APT phase. It is also important to keep in mind that a considerable amount of REC can be expected following APT as indicated in the present and other studies^20,21^.

Among the limitations of this study are the retrospective nature of this project (various clinicians performing therapy, maintenance and periodontal parameter collection in a school setting) and the limited follow-up period. Only compliant patients receiving active and supportive periodontal treatment were included, which limits the external validity of the results. The limited sample size, especially in the OFD group is another limitation of this study, which might explain why more factors were found to significantly affected the clinical outcomes obtained in the SRP versus the OFD group. Overall, the infrabony defects treated in this study were associated with deep PD, infrabony defects with shallower PD might respond differently to SRP and OFD. Prospective clinical studies with long follow-up periods should be conducted to confirm the present findings.

## Conclusion

Within the limitations of the present study, the following conclusions could be drawn: (1) Non-surgical and surgical mechanical root debridement alone led to significant moderate PD reduction, however, this occurred mainly as a result of REC; (2) The frequency of maintenance appointments, the association with furcation defects, as well as the PD and CAL obtained following APT influenced the survival of teeth associated with infrabony defects; (3) Factors such as baseline CAL and PD, and the association with furcation defects significantly affected the short-term clinical outcomes of surgical and non-surgical mechanical root debridement.

## Supplementary Information

Supplementary Information 1.

Supplementary Information 2.

Supplementary Information 3.

Supplementary Information 4.

Supplementary Information 5.
