# Supplementary material for: The effect of non-surgical and surgical mechanical root debridement on infrabony defects: a retrospective study
Source: Sci Rep. 2021 Oct 6;11:19856. doi: 10.1038/s41598-021-99205-z (PMC8494931; doi:10.1038/s41598-021-99205-z)
Supplement: Supplementary file 1 — Supplementary Information 1. [file 41598_2021_99205_MOESM1_ESM.docx]

**The Effect of Non-surgical and Surgical Mechanical Root Debridement on Infrabony Defects: A Retrospective Study**

Jad Majzoub ^1^, BDS, Ali Salami ^2^, MS, PhD, Shayan Barootchi ^1^, DMD, Lorenzo Tavelli ^1,3^, DDS,

Hsun-Liang Chan ^1^, DDS, MS, Hom-Lay Wang ^1*^, DDS, MS, PhD

**Supplementary Figure S1.** Flowchart diagram displaying the patient file screening process leading to the final inclusion of the selected patients.

Records retrieved and screened after the initial search **(n=683)**

GTR with resorbable membranes **(n=** **﻿175)**

GTR using barrier membranes alone without bone graft material **(n=6)**

GTR with non-resorbable membranes **(n=123)**

Root resection cases **(n=136)**

Records with less than 1 year follow up **(n=33)**

Use of bone graft material alone **(n=78)**

Records included in the analysis **(n=132)**
