# Supplementary material for: The effect of non-surgical and surgical mechanical root debridement on infrabony defects: a retrospective study
Source: Sci Rep. 2021 Oct 6;11:19856. doi: 10.1038/s41598-021-99205-z (PMC8494931; doi:10.1038/s41598-021-99205-z)
Supplement: Supplementary file 3 — Supplementary Information 3. [file 41598_2021_99205_MOESM3_ESM.docx]

**The Effect of Non-surgical and Surgical Mechanical Root Debridement on Infrabony Defects: A Retrospective Study**

Jad Majzoub ^1^, BDS, Ali Salami ^2^, MS, PhD, Shayan Barootchi ^1^, DMD, Lorenzo Tavelli ^1,3^, DDS,

Hsun-Liang Chan ^1^, DDS, MS, Hom-Lay Wang ^1*^, DDS, MS, PhD

**Supplementary Table S2.** Characteristics of the included patients and defects at baseline.

| **Characteristics** | **OFD (n = 40)** | **SRP (n = 92)** | **P-value** |
| --- | --- | --- | --- |
| **Males** *n* (%) | 21 (52.5) | 43 (46.7) | 0.543 |
| **Age** | 53.35 ± 11.88 | 51.90 ± 15.26 | 0.558 |
| **Diabetics** *n* (%) | 6 (15.0) | 5 (5.4) | 0.068 |
| **Smoker** *n* (%) | 14 (35.0) | 28 (30.4) | 0.605 |
| **Classification of periodontal disease** | | | |
| ﻿Stage 3 grade A periodontitis *n* (%)^1^ | 3 (7.5) | 8 (8.7) | 0.808 |
| Stage 3 grade B periodontitis *n* (%)^1^ | 2 (5) | 27 (29.3) | <0.001 |
| Stage 3 grade C periodontitis *n* (%)^1^ | 17 (42.5) | 24 (26.1) | 0.041 |
| Stage 4 grade A periodontitis *n* (%)^1^ | 2 (5) | 11 (12) | 0.09 |
| Stage 4 grade B periodontitis *n* (%)^1^ | 8 (20) | 9 (9.8) | 0.068 |
| Stage 4 grade C periodontitis *n* (%)^1^ | 8 (20) | 13 (14.1) | 0.303 |
| **Maintenance per year** | 1.97 ± 0.73 | 1.95 ± 0.58 | 0.759 |
| **Baseline Clinical attachment level (CAL [mm])** | 10.08 ± 1.73 | 8.78 ± 2.07 | 0.001 |
| **Baseline Pocket depth (PD [mm])** | 9.65 ± 1.54 | 8.11 ± 1.63 | <0.001 |
| **Baseline Recession (REC [mm])** | 0.43 ± 1.22 | 0.67 ± 1.51 | 0.412 |
| **Clinical attachment level** (CAL [mm]) | 0.08 ± 1.76 | 0.13 ± 1.829 | 0.860 |
| **Pocket depth** (PD [mm]) | 2.23 ± 1.64 | 1.42 ± 1.71 | 0.010 |
| **Recession** (REC [mm]) | 2.15 ± 1.33 | 1.29 ± 1.56 | 0.001 |
| **Teeth with Degree I furcation involvement** | 10 | 4 | <0.001 |
| **Teeth with Degree II furcation involvement** | 1 | 1 | 0.317 |

Values are arithmetic mean ± SD for continuous variables.

Categorical variables are shown as numbers (*n*) and percentages (%). *n*: sample size.

REFERENCES

1 Tonetti, M. S., Greenwell, H. & Kornman, K. S. Staging and grading of periodontitis: Framework and proposal of a new classification and case definition. *J Periodontol* **89 Suppl 1**, S159-S172, doi:10.1002/JPER.18-0006 (2018).
