# Supplementary material for: The effect of non-surgical and surgical mechanical root debridement on infrabony defects: a retrospective study
Source: Sci Rep. 2021 Oct 6;11:19856. doi: 10.1038/s41598-021-99205-z (PMC8494931; doi:10.1038/s41598-021-99205-z)
Supplement: Supplementary file 4 — Supplementary Information 4. [file 41598_2021_99205_MOESM4_ESM.docx]

**The Effect of Non-surgical and Surgical Mechanical Root Debridement on Infrabony Defects: A Retrospective Study**

Jad Majzoub ^1^, BDS, Ali Salami ^2^, MS, PhD, Shayan Barootchi ^1^, DMD, Lorenzo Tavelli ^1,3^, DDS,

Hsun-Liang Chan ^1^, DDS, MS, Hom-Lay Wang ^1*^, DDS, MS, PhD

**Supplementary Table S3.** Life table descriptively summarizing the survival probabilities according to the Kaplan-Meier analysis.

| **OFD** | | | | | **95% Confidence Interval** | |
| --- | --- | --- | --- | --- | --- | --- |
| **Time (months)** | **Number at risk** | **Number of event(s)** | **Survival** | **SE** | **Lower bound** | **Upper bound** |
| 0 | 39 | 0 | 1.00 | - | - | - |
| 36 | 33 | 1 | 0.971 | 0.029 | 0.914 | 1.027 |
| 54 | 28 | 1 | 0.937 | 0.043 | 0.853 | 1.022 |
| 55 | 26 | 1 | 0.902 | 0.054 | 0.797 | 1.008 |
| 67 | 23 | 1 | 0.865 | 0.063 | 0.741 | 0.989 |
| 81 | 14 | 1 | 0.807 | 0.081 | 0.648 | 0.966 |
| 83 | 12 | 1 | 0.745 | 0.096 | 0.557 | 0.933 |
| 87 | 10 | 2 | 0.621 | 0.113 | 0.399 | 0.843 |
| 90 | 8 | 1 | 0.552 | 0.120 | 0.317 | 0.787 |
| 96 | 6 | 1 | 0.473 | 0.126 | 0.226 | 0.720 |
| 120 | 3 | 1 | 0.355 | 0.139 | 0.082 | 0.628 |
| 125 | 2 | 1 | 0.237 | 0.134 | -0.026 | 0.499 |
| 156-240 | 1 | 1 | 0.118 | 0.107 | -0.092 | 0.328 |
| **SRP** | | | | | **95% Confidence Interval** | |
| **Time (months)** | **Number at risk** | **Number of event(s)** | **Survival** | **SE** | **Lower bound** | **Upper bound** |
| 0 | 91 | 0 | 1.00 | - | - | - |
| 34 | 65 | 1 | 0.985 | 0.015 | 0.955 | 1.014 |
| 43 | 60 | 1 | 0.969 | 0.022 | 0.926 | 1.011 |
| 56 | 52 | 1 | 0.950 | 0.028 | 0.896 | 1.005 |
| 58 | 50 | 1 | 0.932 | 0.033 | 0.867 | 0.997 |
| 63 | 46 | 1 | 0.912 | 0.038 | 0.838 | 0.986 |
| 81 | 39 | 1 | 0.889 | 0.043 | 0.804 | 0.974 |
| 85 | 30 | 1 | 0.860 | 0.050 | 0.762 | 0.959 |
| 89 | 21 | 1 | 0.821 | 0.061 | 0.701 | 0.942 |
| 91 | 19 | 1 | 0.780 | 0.071 | 0.642 | 0.919 |
| 92 | 15 | 4 | 0.616 | 0.092 | 0.436 | 0.796 |
| 98 | 11 | 1 | 0.565 | 0.098 | 0.373 | 0.756 |
| 102 | 9 | 1 | 0.508 | 0.103 | 0.307 | 0.710 |
| 115 | 6 | 1 | 0.436 | 0.111 | 0.218 | 0.653 |
| 126 | 4 | 1 | 0.349 | 0.118 | 0.117 | 0.580 |
| 132 | 3 | 1 | 0.261 | 0.116 | 0.033 | 0.489 |
| 153-192 | 1 | 1 | 0.131 | 0.109 | -0.083 | 0.345 |
