# Supplementary material for: The effect of non-surgical and surgical mechanical root debridement on infrabony defects: a retrospective study
Source: Sci Rep. 2021 Oct 6;11:19856. doi: 10.1038/s41598-021-99205-z (PMC8494931; doi:10.1038/s41598-021-99205-z)
Supplement: Supplementary file 5 — Supplementary Information 5. [file 41598_2021_99205_MOESM5_ESM.docx]

**The Effect of Non-surgical and Surgical Mechanical Root Debridement on Infrabony Defects: A Retrospective Study**

Jad Majzoub ^1^, BDS, Ali Salami ^2^, MS, PhD, Shayan Barootchi ^1^, DMD, Lorenzo Tavelli ^1,3^, DDS,

Hsun-Liang Chan ^1^, DDS, MS, Hom-Lay Wang ^1*^, DDS, MS, PhD

**Supplementary Table S4.** Results of the multilevel cox proportional hazard models evaluating the effect of different variables (excluding the association with furcation defects) on the survival of the teeth treated with SRP alone.

|  | **Univariate analysis** | | | | **Multivariate analysis** | | | |
| --- | --- | --- | --- | --- | --- | --- | --- | --- |
| **Variable** | **HR** | **Std. Error** | **95% CI** | **P-value** | **HR** | **Std. Error** | **95% CI** | **P-value** |
| Age | 1.014 | 0.019 | (0.978, 1.053) | 0.447 |  |  |  |  |
| Gender (male) | 0.887 | 0.468 | (0.354, 2.219) | 0.797 |  |  |  |  |
| Smoking | 1.607 | 0.478 | (0.630, 4.102) | 0.321 |  |  |  |  |
| Diabetes | 1.824 | 0.761 | (0.410, 8.111) | 0.430 |  |  |  |  |
| **Maintenance per year** | **0.224** | **0.523** | **(0.081, 0.625)** | **0.004** | **0.235** | **0.514** | **(0.086, 0.644)** | **0.005** |
| Initial PD | 0.810 | 0.200 | (0.547, 1.200) | 0.293 |  |  |  |  |
| Initial CAL | 1.008 | 0.121 | (0.796, 1.277) | 0.947 |  |  |  |  |
| **Final PD** | **1.444** | **0.160** | **(1.056, 1.975)** | **0.021** | **1.407** | **0.156** | **(1.037, 1.910)** | **0.028** |
| Final CAL | 1.163 | 0.127 | (0.907, 1.492) | 0.234 |  |  |  |  |

The values in bold signifies statistical significance; CI, confidence intervals. Data related to the presented variables was available for all 132 subjects
